# DNA Adductomics: A Narrative Review of Its Development, Applications, and Future

**DOI:** 10.3390/biom14091173

**Published:** 2024-09-19

**Authors:** Mengqiu Cao, Xinyu Zhang

**Affiliations:** School of Public Health, Hongqiao International Institute of Medicine, Shanghai Jiao Tong University School of Medicine, Shanghai 200025, China; chizhuzhu@sjtu.edu.cn

**Keywords:** DNA adductomics, LC-MS/MS, detection, biomarkers

## Abstract

DNA adductomics is the global study of all DNA adducts and was first proposed in 2006 by the Matsuda group. Its development has been greatly credited to the advances in mass spectrometric techniques, particularly tandem and multiple-stage mass spectrometry. In fact, liquid chromatography-mass spectrometry (LC-MS)-based methods are virtually the sole technique with practicality for DNA adductomic studies to date. At present, DNA adductomics is primarily used as a tool to search for DNA adducts, known and unknown, providing evidence for exposure to exogenous genotoxins and/or for the molecular mechanisms of their genotoxicity. Some DNA adducts discovered in this way have the potential to predict cancer risks and/or to be associated with adverse health outcomes. DNA adductomics has been successfully used to identify and determine exogenous carcinogens that may contribute to the etiology of certain cancers, including bacterial genotoxins and an *N*-nitrosamine. Also using the DNA adductomic approach, multiple DNA adducts have been observed to show age dependence and may serve as aging biomarkers. These achievements highlight the capability and power of DNA adductomics in the studies of medicine, biological science, and environmental science. Nonetheless, DNA adductomics is still in its infancy, and great advances are expected in the future.

## 1. Introduction

Science is advancing toward comprehensiveness. Traditionally, in a complex system, a scientist selects an individual component element and then investigates the element carefully. Sometimes, a limited number of other elements that are related to a specific one, and the interactions among these elements are investigated together. This “one thing at a time” approach has provided the majority of our current knowledge. However, with such an approach, it is almost impossible to get a good understanding of the system. To address the issue, scientists have been taking a different approach, that is, investigating all component elements in a system at a time.

Take protein research as an example. A cell contains tens of thousands of different proteins; however, the traditional approach investigates only one protein or at most a few proteins at a time. Apparently, we cannot see the entirety as we are limited to a tiny fraction of cellular proteins and overlook the bigger picture. The situation is exactly what the idiom “cannot see the forest for the trees” means. Realizing the limitation of the traditional approach, researchers strive to “see the forest” through globally investigating all proteins at a time, that is, the proteomic approach.

In scientific research, the trend toward comprehensiveness, accompanied by the advances in techniques, has led to the emergence of a new approach, i.e., globally investigating all component elements in a system, in almost every sub-field of biological science, biomedical research, and environmental science in the past three decades, such as genomics, epigenomics, transcriptomics, proteomics, metabolomics, interactomics, immunomics, microbiomics, exposomics, etc. [1,2,3,4,5,6,7]. The new approach is called “omics”. Simply put, omics, which is usually used as a suffix added to the ends of particular words, is the study of the entire set of molecules or molecular processes in a given system at a particular level. Accordingly, the suffix “-ome” refers to the totality of molecules or molecular processes in a given system. Thus, DNA adductome, coined by Kanaly et al. in 2006 [8], refers to the totality of all DNA adducts in the target investigated, which can be cells, tissues, and organisms, and DNA adductomics is the global study of DNA adducts in a particular target. 

**Figure 1:**
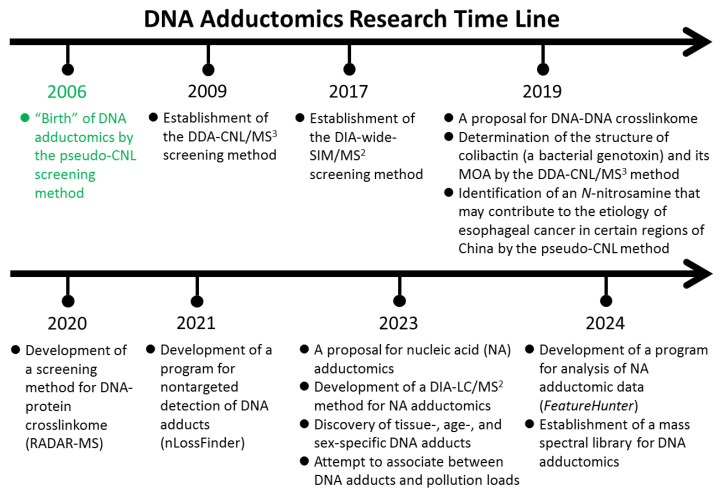
DNA adductomics research timeline.

DNA adductomics is a relatively new addition to the omics world, and the studies of DNA adductomics have been limited to date; a search using “DNA adductomics” in PubMedline produces only ~110 articles. The establishment and development of the field are greatly credited to the advances in mass spectrometric techniques, particularly tandem mass spectrometry (MS/MS) and multiple-stage mass spectrometry (MS^n^). From 2006 to 2020, many studies have been dedicated to the development of the mass spectrometric methodology and related data processing. A research timeline with the important advances in DNA adductomics is shown in Figure 1.

At present, DNA adductomics is mostly used as a tool to screen for DNA adducts, known or unknown, providing important evidence for exposure to specific carcinogens and/or related consequences. As a result, it is necessary to first discuss DNA adducts. Thus, in the article, we first provide a brief overview of DNA adducts and discuss their classifications, and then review the development in the methodologies of DNA adductomics. Screening for DNA crosslinks and apurinic/apyrimidinic (AP) sites, the “products” of DNA adducts, is discussed separately. Subsequently, we review the cases of how DNA adductomics contributes to advances in medicine, biological science, and environmental science. Lastly, we provide our perspectives on future developments.

## 2. DNA Adducts and Their Classifications

### 2.1. A Brief Overview of DNA Adducts

DNA adducts are formed when DNA undergoes chemical modifications. Usually, such chemical modifications are attributed to the attack on DNA by reactive chemical species, although UV irradiation is capable of directly inducing DNA chemical modifications through photoreactions, forming cyclobutane pyrimidine dimers (CPD) and (6-4) pyrimidine–pyrimidone photoproducts [9]. The modifications of DNA can occur on the nucleobases, 2′-deoxyribose (dR) moieties, and phosphate groups, although in most cases the modifications on the nucleobases are the dominant form [10,11]. Nucleobases contain multiple nitrogen and oxygen atoms, which are electron-rich and thus vulnerable to attack by electrophiles [10].

DNA adducts are a form of DNA damage; if not repaired in time, they can interfere with DNA transcription and replication and induce mutations, contributing to carcinogenesis [12]. Thus, it is generally accepted that the formation of DNA adducts is the initial molecular event in carcinogenesis. Not surprisingly, most studies of DNA adducts are related to genotoxicity and mutagenicity/carcinogenicity. 

DNA adducts are important biomarkers. Their structures provide critical information about the reactive chemical species that directly attack DNA, thus helping identify the culprits that contribute to carcinogenesis, as evidenced by two excellent examples [13,14]. In addition, the formation of DNA adducts is a significant driver of disease onset and progression through mutagenesis; thus, DNA adducts can be used as biomarkers to predict, prevent, and diagnose diseases [15]. Such a case is 7-(deoxyadenosin-*N*^6^-yl)aristolactam I, a DNA adduct of aristolochic acid, which is clearly associated with urothelial carcinoma of the upper urinary tract and has been used as a biomarker of cancer risk [16]. Oral cell DNA adducts of carcinogens in cigarette smoke are potential biomarkers for lung cancer susceptibility in smokers [17]. 

### 2.2. Classifications of DNA Adducts

DNA adducts can be categorized into monoadducts and crosslinks. The former are the adducts formed through chemical modifications by small molecules on individual nucleobases. However, if the modifications of nucleobases occur by reacting with macromolecules, such as proteins and RNA, the adducts formed are classified as crosslinks, i.e., DNA-protein crosslinks (DPCs) and DNA-RNA crosslinks, respectively [18,19,20]. DNA can also be linked to itself, forming DNA-DNA crosslinks, which include interstrand and intrastrand crosslinks [21,22]. 

It is an interesting issue whether epigenetic modifications of DNA should be considered DNA adducts. Theoretically, pristine DNA is a double-stranded polynucleotide, with all nucleobases being unmodified. Thus, by the definition of DNA adducts, epigenetic modifications should be classified as DNA adducts. It may cause some confusion because the formation of DNA adducts is usually accepted as a type of damage to DNA, whereas epigenetic modifications are a naturally biological process that serves the regulation of gene expression. However, the boundary between DNA adducts as a form of DNA damage and epigenetic modifications is actually blurred. On the one hand, many epigenetically modified nucleobases often have the origins from reactions with reactive chemical species. For example, 5-hydroxymethyl-2′-deoxycytidine (5-hmdC), 5-formyl-2′-deoxycytidine (5-fdC), 5-carboxyl-2′-deoxycytidine (5-cadC), and 5-hydroxymethyl-2′-deoxyuridine (5-hmdU) are epigenetic marks but can also be produced from reactive oxygen species (ROS) [10]. On the other hand, 8-oxo-2′-deoxyguanosine (8-oxo-dG), a major product of DNA oxidation and a widely-used oxidative stress biomarker, also serves as an epigenetic mark that affects transcriptional regulatory elements and other epigenetic modifications [23,24].

Another issue concerns AP sites, which are the sites in DNA without nucleobases and thus are also called the abasic sites [25]. The sites, by definition, are certainly not DNA adducts; however, they are generated as the result of the formation of DNA monoadducts. The formation of DNA monoadducts triggers base excision repair (BER); the first step of BER is the excision of damaged nucleobases by glycosylases, producing AP sites [26]. Thus, AP sites are the intermediates during the DNA repair process. In addition, alkylation of some endocyclic nitrogens on nucleobases, including N7G, N7A, N3G, N3A, N1A, and N3C, destabilizes nucleobases, facilitating spontaneous deglycosylation and the formation of AP sites [27]. In a sense, AP sites are the “products” of DNA adducts. Therefore, in the review, AP sites will be discussed as well.

## 3. Detection of DNA Adducts and DNA Adductomic Methodologies

The detection of all DNA adducts in the target investigated is at the core of the studies of DNA adductomics. However, it needs to be noted that DNA adducts include monoadducts and crosslinks, and most methodologies have been developed with a focus on the detection of monoadducts. Crosslinks, particularly DPCs, are usually neglected in a vast majority of the studies, which is primarily caused by the fact that the detection of DPCs requires different methodologies [28]. On the other hand, DNA-DNA and DNA-RNA crosslinks can be detected using the methodologies to screen for monoadducts, but some modifications in the methodologies are needed [20,29]. So far, no methodologies that can truly detect all DNA adducts have been developed.

### 3.1. Detection of DNA Monoadducts

#### 3.1.1. Liquid Chromatography-Mass Spectrometry (LC-MS)-Based Methodologies

At present, LC-MS/MS and LC-MS^n^ are the mainstream techniques to detect DNA adducts. They provide important structural information about analytes, including molecular weights, chemical compositions, and the identities of nucleobases. In the instrumental configurations, LC can be coupled with low-resolution or high-resolution (HR) mass spectrometers. HR instruments are significantly advantageous over their low-resolution counterparts; through accurate mass monitoring, LC-HR MS/MS or LC-HR MS^n^ can help determine the chemical compositions of analytes and greatly increase the specificity of the adduct identification.

**Figure 2:**
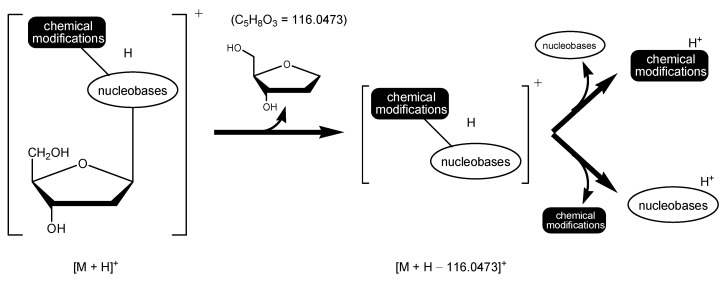
The principle of the MS-based methodologies for global detection of DNA adducts.

The principle of the LC-MS-based methodologies for global detection of DNA adducts is rooted in a universal observation: in MS/MS, the ions of 2′-deoxynucleosides readily lose a neutral fragment of dR (mass 116, or 116.0473 in HRMS) when subjected to collision-induced dissociation (CID), generating the product ions [M + H − 116]^+^ or [M + H − 116.0473]^+^ (Figure 2). Therefore, DNA adducts are detected through monitoring the ion transitions [M + H]^+^ → [M + H − 116]^+^ or [M + H − 116.0473]^+^. Further fragmentation of the product ions in MS^3^ can lead to neutral loss of either nucleobases or the chemical modification moieties (Figure 2), generating the ions of the chemical modification moieties or nucleobases and thus providing more robust evidence for the identities of DNA adducts.

The screening for DNA adducts in mass spectrometers can be performed under different scan modes, such as the constant neutral loss (CNL) mode [30] and the multiple reaction monitoring (MRM) mode [8]. However, we will not describe the technical details because they have been discussed in depth in several excellent reviews [31,32,33,34,35]. Rather, we will only review the development in the screening methods briefly as below.

Depending on the scan modes and the instrumental configurations, several DNA adductomic screening methods have been developed. The simplest one is the pseudo-CNL screening method, which was established by the Matsuda laboratory in their study of pioneered DNA adductomics [8]. The method, which is established on the MRM scan mode, consists of a consecutive series of MRM transitions covering a wide *m*/*z* range (e.g., from 228.8 to 602.8 in their study [8]), with the MRM transitions being set to monitor the neutral loss of dR. The method provides a basic technical means for studies of DNA adductomics because it can be run on low-end triple quadrupole (QqQ) mass spectrometers, which are popular and easily accessible for most laboratories. In fact, the researchers in the Matsuda laboratory kept using the method in a series of subsequent studies [36,37,38,39,40,41,42]. When running on HR mass spectrometers, the method has a higher specificity and can provide accurate molecular weights of DNA adducts, greatly facilitating the identification of DNA adducts. The successful use of the pseudo-CNL screening method running on HRMS was showcased in a series of studies conducted by the Totsuka laboratory [13,43,44], in particular, the successful detection of a nitrosamine-derived DNA adduct in esophageal cancer patients that provides critical information for the identification of the likely environmental carcinogen responsible for esophageal cancer [13]. On the other hand, the Chao laboratory tested the CNL scan mode on a QqQ instrument and found that the sensitivity was ~10-fold lower than the pseudo-CNL screening method operating on the same instrument [30].

Another DNA adductomic screening method utilizes data-dependent CNL scan mode followed by triple-stage mass spectrometry (MS^3^) and is called the data-dependent acquisition constant neutral loss-triple stage mass spectrometry (DDA-CNL-MS^3^) screening method, which was developed first by the Turesky laboratory on a two-dimensional linear quadrupole ion trap mass spectrometer (LIT/MS) [45], and was subsequently used to search for DNA adducts of a natural product [46]. Like the pseudo-CNL screening method, the HR instruments dramatically increase the specificity of the adduct identification [47]. In addition, the neutral loss of the four nucleobase moieties (guanine, 151.0494; adenine, 135.0545; thymine, 126.0429; cytosine, 111.0433) can be integrated into the method to detect the DNA adducts that spontaneously lose the dR moieties [48]. The method was successfully applied to detect two unknown DNA adducts induced by colibactin, a small-molecule genotoxin that is produced by certain *Escherichia coli* strains residing in the human gut and may contribute to colorectal cancer pathogenesis [14]. The Balbo laboratory further refined the DDA-CNL-MS^3^ screening method to incorporate the detection of endogenous DNA adducts, most of which are hydrophilic and have similar masses and chromatographic behaviors to the unmodified nucleosides and ubiquitous background signals [49], and to improve the detection of low levels of DNA adducts [50].

The third major method is called the wide-selected ion monitoring tandem mass spectrometry (wide-SIM/MS^2^) screening method, which was developed by the Turesky laboratory in 2017 [51]. It is a data-independent acquisition (DIA) method and can be used in a targeted or untargeted fashion [51]. Compared to the DDA-CNL-MS^3^ method, the advantage of the DIA-wide-SIM/MS^2^ approach is the completeness of the analysis [33]. Moreover, the performance of the DIA-wide-SIM/MS^2^ method is also superb; a comparison among targeted DNA adductomic methodologies of Orbitrap-based DIA-wide-SIM/MS^2^ and DDA-CNL/MS^3^ analyses and QqQ-based CNL and pseudo-CNL analyses indicated that the performance of the four approaches was DIA-wide-SIM/MS^2^ > DDA-CNL/MS^3^ > pseudo-CNL > CNL on the basis of the numbers of adducts detected in a sample of calf thymus DNA spiked 15 adduct standards. The comparison was carefully carried out using the same sample with identical chromatography and ion source conditions, and the numbers of adducts detected were 12, 7, 2, and 0 for the four approaches, respectively, out of 15 at the lowest levels of spiking (4–8 adducts per 10^9^ nucleotides) [33,51]. The DIA-wide-SIM/MS^2^ method was subsequently applied to examine nontumor bladder tissues from bladder cancer patients, and many DNA adducts of aromatic amines and lipid peroxidation (LPO) products were detected [52].

In addition to the above pseudo-CNL, DDA-CNL-MS^3^, and DIA-wide-SIM/MS^2^ approaches, other laboratories also established their own versions of the DNA adductomic screening methods, which were based on the same principle of the neutral loss of dR and nucleobases but used different scanning modes and/or instrumental configurations. For example, the Vanhaecke laboratory established a method using the full scan, SIM, and SIM-MS/MS modes [53]. It should be noted that the Motwani laboratory developed a DIA-LC/MS^2^ method for simultaneous global screening for DNA and RNA adducts in a single run [54].

An important issue in the LC-MS-based adductomic methodologies is the data processing and analysis, which are a great challenge because the DNA adductomic screening methods produce large amounts of data [55]. Specific software tools and algorithms for data processing are necessary [55], and a mass spectral database of DNA adducts needs to be established [56]. Several laboratories have been working on the issues and have made great advances. To analyze the data generated from the DIA-wide-SIM/MS^2^ screening method, Walmsley et al. developed a software called wSIM-City [57]. Similarly, to process the data obtained from the DDA-CNL-MS^3^ screening method, the Balbo laboratory developed an automated workflow utilizing a new feature detection algorithm called DFBuilder [58]. In 2021, the Motwani laboratory developed a graphical user interface program, called nLossFinder, in the MATLAB platform for the nontargeted detection of DNA adducts [59]. Due to a conceptual extension from DNA adductomics to nucleic acid (NA) adductomics, which includes all modifications of DNA and RNA [60], the Chao laboratory recently developed a software called “*FeatureHunter*”, which provides the automated extraction, annotation, and classification of different types of key NA modifications based on the MS and MS/MS spectra, using a user-defined feature list [61]. For databases, La Barbera et al. established one with 582 entries that is publicly available (https://gitlab.com/nexs-metabolomics/projects/dna_adductomics_database, accessed on 9 September 2024) [62]. Recently, through collecting synthetic DNA adduct standards (~500) from the research community and acquiring their MS^n^ (n = 2, 3) fragmentation spectra, Walmsley et al. created the most comprehensive collection of DNA adduct fragmentation spectra available to date (https://sites.google.com/umn.edu/dnaadductportal, accessed on 9 September 2024) [63]. 

#### 3.1.2. Raman Spectroscopy

In the detection of DNA adducts, Raman spectroscopy is a potentially promising technique. Raman spectroscopy is based on Raman scattering and is capable of identifying unknown molecules by measuring their vibrational fingerprints in a nondestructive fashion [64].

Raman spectroscopy was used to investigate DNA early on; to our best knowledge, the earliest study dated back more than 50 years ago. In 1968, Hirano first reported the Raman spectra of DNA in aqueous solution [65]. Since then, much research has been conducted, and Raman spectroscopy was soon used to investigate the interaction of antitumor drugs with DNA. In 1975, Chinsky et al. reported such a study between actinomycin D, an anticancer drug, and DNA [66]. However, actinomycin D exerts its antitumor effects through intercalating DNA and inhibiting RNA transcription without causing the formation of DNA adducts. 

Mansy and Peticolas may be the first to investigate DNA adducts using Raman spectroscopy [67]. In a study published in 1976, they compared the Raman spectra of poly(dG)·poly(dC) with and without treatment by mustards and observed significant differences in a few bands. The differences were assigned as the features of alkylation at the *N*7 position of guanine, which was supported by using *N*7-methylguanosine as a model compound [67].

However, Raman scattering is extremely weak, thus limiting its applications until the discovery of surface-enhanced Raman spectroscopy (SERS) in the mid-1970s. The million-fold enhancement of Raman scattering on metal surfaces makes many applications in biology possible, and the single-molecule sensitivity has been demonstrated [64]. In 1984, Séquaris et al. used SERS to investigate the interactions of DNA with chlorodiethylenetriaminoplatinum (II) chloride ([Pt(dien)Cl]Cl) and *cis*-Pt(NH_3_)_2_Cl_2_, two platinum derivatives that can form DNA monoadducts and crosslinks, respectively [68]. However, the study focused on the stereochemical configurations of Pt-DNA complexes rather than the detection of DNA adducts. Afterward, other researchers similarly investigated the interactions of DNA with a variety of genotoxins, including mitomycin C, chloroacetaldehyde, bleomycin, carboplatin, cisplatin, and chloroethyl nitrosourea derivatives [69,70,71,72,73,74,75,76,77].

In a proof-of-concept study published in 2011, Barhoumi and Halas demonstrated the feasibility of using SERS to detect methylation of adenine, methylation, and hydroxymethylation of cytosine, and guanine oxidation (i.e., 8-oxo-dG) [78]. In 2015, Guerrini et al. reported the first examples of SERS recognition of single base mismatches and base methylations (5-methylated cytosine and *N*^6^-methylated adenine) in DNA duplexes at nanogram DNA levels [79]. Subsequently, the researchers further reported the identification and relative quantification of 4 different cytosine modifications (5-methylcytosine, 5-hydroxymethylcytosine, 5-hydroxycytosine, and 5-bromocytosine) in single- and double-stranded DNA by using SERS spectroscopy [80]. 

#### 3.1.3. Other Methods

In 2021, a method called CAX-Prelabeling was reported, which utilizes the tendency of DNA adducts to distort/destabilize the local structures of dsDNA into what are sometimes referred to as “DNA bubbles” [81]. The formation of DNA bubbles renders the regions of DNA and the resident adducts more susceptible to further reaction, as with an alkylating mass tag. Thus, the researchers selected *N*-(2-(bromomethyl)benzyl)-*N*,*N*-diethylethanaminium bromide (CAX-B) as the mass tag to label DNA adducts. After labeling, DNA is enzymatically digested into nucleotides, which are subjected to LC-MS/MS analysis. The authors tested the method using calf thymus DNA treated with different electrophiles and successfully detected the anticipated DNA adducts with good sensitivities [81].

### 3.2. Detection of DNA Crosslinks and AP Sites

#### 3.2.1. DNA-DNA and DNA-RNA Crosslinks

DNA-DNA and DNA-RNA crosslinks can be detected through LC-MS/MS, similar to the detection of monoadducts, although the detection methods need some modifications [20,48]. Utilizing the features of DNA-DNA crosslinks, Hu et al. developed a method specifically for their comprehensive and untargeted detection, which was coined as “DNA crosslinkomics” [29]. The method identifies DNA-DNA crosslinks by monitoring the accurate mass neutral loss of one dR moiety (116.0474) and also two dR ones (232.0948), and of one nucleobase moiety as well as any two nucleobases (e.g., 302.0988 for guanine-guanine and 286.1038 for guanine-adenine). Using the method, the authors examined calf thymus DNA treated with formaldehyde and chlorambucil, a nitrogen mustard to be used as a chemotherapy drug, and identified both known and unknown DNA-DNA crosslinks [29]. On the other hand, so far there has been only one report on the detection of DNA-RNA crosslinks; in 2022, Dator et al. used an improved HR segmented full scan data-dependent neutral loss MS^3^ screening strategy to discover DNA-RNA crosslinks for the first time [20]. The formaldehyde-induced DNA-RNA crosslinks were detected in the forms of 2′-deoxyadenosine (dA)-CH_2_-guanosine and dG-CH_2_-guanosine through characteristic fragment ions including adenine·H^+^, guanine·H^+^, and guanosine·H^+^ [20].

#### 3.2.2. DPCs

DPCs can be detected through a few different techniques, including the comet assay, fluorescence labeling, immunodetection, and LC-MS/MS. However, like DNA monoadducts and DNA-DNA crosslinks, LC-MS/MS is the major technique for global detection of DPCs. 

In some sense, global detection of DPCs should be considered to be a subcategory of proteomics rather than DNA adductomics, because the detection of DPCs is essentially the identification of the proteins adducted to nucleic acids. The LC-MS-based methodology for the global detection of DPCs is actually a proteomic approach, in which DPCs are digested with specific proteases, and the identities of proteins are determined through the resulted peptides by LC-MS/MS analysis. These have been reviewed before [28].

DPCs can also be detected through fluorescence labeling and immunodetection [82,83], although it is not clear whether these methods can globally detect all DPCs. Shoulkamy et al. reported the detection of DPCs by direct fluorescence labeling; in their method, DPCs were labeled with fluorescein isothiocyanate (FITC) and quantified by fluorometry or western blotting using anti-FITC antibodies [82]. The Maizels laboratory developed a quantitative immunodetection method termed RADAR (rapid approach to DNA adduct recovery) assay, which was sufficiently sensitive to detect topoisomerase 1-DNA crosslink in as little as 60 ng of DNA, corresponding to 10,000 human cells [83]. The assay was subsequently improved by incorporating an ELISA assay, increasing the applicability of the assay [84]. In 2020, Kiianitsa and Maizels improved the assay again by using MS to identify proteins, thus converting the original assay to an adductomics tool, because MS enables unbiased identification of proteins independent of the antibodies, which may be limited in specificity or unable to detect adducts undergoing proteolytic repair that eliminates epitopes critical for antibody recognition [85]. Using the method, they found that in normally proliferating human cells, the repertoire of adducted proteins—the “adductome”—is comprised of a limited number of proteins belonging to specific functional groups and that it is greatly enriched for histones, HMG proteins, and proteins involved in RNA splicing. These DPCs are the cellular background and are considered to be induced by endogenous formaldehyde [85].

#### 3.2.3. AP Sites

AP sites are ubiquitous DNA lesions, which stem from DNA repair via BER and spontaneous depuration of DNA [25,26,27]. 8-Oxo-dG is repaired primarily through the BER pathway and thus is one of the major sources of AP sites [23,24]. Indeed, the levels of AP sites increase under oxidative stress conditions [86]. In human leukocytes, AP sites have been observed to increase significantly with subject age in nonsmokers, which implicates an increase in endogenous oxidative insults and a decrease in AP site repair [87].

Based on the latest research, the basal level of AP sites, as measured in the liver and kidney of rats, is ~1 site per 10^7^ nucleotides, and oxidative stress induced by ferric nitrilotriacetate causes an almost 4-fold increase in the level of AP sites in rat liver [86]. The levels measured in human leukocytes are 2.10 ± 0.59 and 2.07 ± 0.60 sites per 10^7^ nucleotides for nonsmokers and smokers, respectively, with no difference being observed [87].

AP sites have been investigated for 5 decades, and many methods have been developed for quantitation of AP sites. A comprehensive review of these methods is beyond the scope of the article. Readers can obtain the information from ref. [86,88] and references cited therein. However, it is worth noting that Raman spectroscopy is also capable of detecting AP sites. In a proof-of-concept study, Guerrini and Alvarez-Puebla demonstrated the first example using SERS to identify AP sites in dsDNA at the single-base level. Notably, SERS provides additional conformational information on the nucleobases opposite to the AP sites (intra- vs. extra-helical) [89].

It should be noted that large numbers of AP sites are generated during sample workup. As a result, most studies in the literature may have significantly overestimated the numbers of AP sites. For example, a review in 2011 provided a value of 30,000 AP sites per cell [90]. However, as demonstrated by Chen et al., most of the AP sites detected in previous studies may be attributed to the artefactual formation during DNA isolation, digestion, or the derivatization step [86]. Indeed, after carefully optimizing their method, the authors quantified the AP sites in the liver and kidney of rats at 0.91 and 1.13 per 10^7^ nucleotides, respectively (~300 AP sites per cell). Even in rats undergone oxidative stress, the data increased to only 3.31 and 1.60 per 10^7^ nucleotides, respectively.

## 4. Applications of DNA Adductome Analysis

DNA adductomics provides a powerful tool to screen for DNA adducts in samples with high sensitivity. DNA adducts carry structural information about the reactive chemical species that attack DNA and are important biomarkers. Thus, DNA adductomics has many applications in medicine, environmental science, and biological science. 

### 4.1. The Identification of DNA Adducts as Potential Predictive Biomarkers

Biomarkers are one of the cornerstones of modern evidence-based medicine [91] and include diagnostic, monitoring, response, predictive, prognostic, safety, and susceptibility/risk biomarkers [15,92]. They can also inform drug discovery and development, dose selection, and trial design [91]. The Lung Cancer Master Protocol (Lung-MAP; S1400), a precision medicine clinical trial launched in 2014 by the U.S. National Cancer Institute (NCI) for people with advanced non-small cell lung cancer that has continued to grow after treatment, is a completed biomarker-driven master protocol and has been found to meet its goal to address unmet needs in the treatment of advanced lung cancers [93].

In oncology, predictive biomarkers provide information about the effects of therapeutic interventions. Most chemotherapeutic drugs kill cancer cells by modifications of DNA, making DNA adducts produced by the drugs natural candidates of predictive biomarkers. DNA adducts are expected to be better biomarkers than metabolites of drugs because adducts reflect DNA damage caused by drugs, whereas metabolites only indicate drug exposure. Therefore, due to its capability to search and discover new DNA adducts, DNA adductomics plays an important role in the studies concerning chemotherapeutic drugs.

Several laboratories have advanced in this direction. A case of such studies is the investigation of the mode of action (MOA) of cyclophosphamide (CPA), a commonly used chemotherapeutic agent [94]. The drug has been known to form DNA monoadducts and crosslinks; however, it has a complex metabolic pathway and can generate several metabolites that are able to interact with DNA. In addition, CPA can also induce the production of ROS. Thus, it is expected that CPA causes the formation of multiple types of DNA adducts, in addition to the adducts that have been discovered. In the meantime, CPA lacks biomarkers that can evaluate the therapeutic effect and toxicity [94], and this is a good case to apply the DNA adductomic approach. Indeed, the researchers used an LC-HR MS^3^ DNA adductomic method to comprehensively profile the reactions of CPA and its metabolites with DNA in vitro and identified 40 DNA adducts, among which 20 adducts were detected in samples from patients undergoing chemotherapy. Most of the 40 adducts, including 29 monoadducts and 11 crosslinks, are new DNA adducts, and several adducts were identified as promising predictive biomarkers. The study is an excellent case to highlight the power and usefulness of DNA adductomic analysis in the search of predictive biomarkers for chemotherapy. The approach was also successfully applied in other chemotherapeutic drugs, such as doxorubicin, busulfan, and CP-506, a newly developed DNA-alkylating hypoxia-activated prodrug [95,96,97]. Although more studies are needed to determine the predictability of the adducts identified in these studies, the discovery of many new DNA adducts lays the foundations for further studies.

Certainly, screening for candidates of predictive biomarkers is not limited to chemotherapeutic drugs; it can also be applied to other carcinogens. Alcohol consumption is known to be a risk factor for several cancers, including cancers of the head and neck and the esophagus. Therefore, it is of great value to search for predictive biomarkers that can reflect the cancer risk. In this regard, Guidolin et al. used a DNA adductomic approach to profile acetaldehyde-DNA adducts, because acetaldehyde, the major metabolite of ethanol, is generally considered to be the culprit of alcohol carcinogenesis [50]. The authors identified 22 DNA adducts, among which 16 adducts had not been reported before. Importantly, 17 out of 22 adducts were detected in DNA isolated from oral cells collected from humans exposed to a dose of alcohol, and their amounts increased significantly after the exposure. Thus, these adducts are potential predicitive biomarkers for alcohol-related cancer risk.

When serving as predictive biomarkers, it is not necessary for DNA adducts to have unambiguous origins. Many DNA adducts originated from endogenously biochemical processes (e.g., oxidative stress) may also have predictive potential for cancer risks. Through comparisons among the DNA adductomes of different tissues of patients, the Japanese groups, led by Matsuda and Sugimura, made efforts to investigate such DNA adducts as predictive biomarkers. The researchers identified several DNA adducts of two LPO products, (2*E*)-4-oxo-2-nonenal (4-ONE) and (2*E*)-4-oxo-2-hexenal (4-OHE) [37]. They found that three adducts derived from 4-ONE, that is, heptanone-etheno-2′-deoxycytidine (H*ε*dC), heptanone-etheno-2′-deoxyadenosine (H*ε*dA), and heptanone-etheno-2′-deoxyguanosine (H*ε*dG), were ubiquitous in human tissues, with the medians being 10, 15, and 8.6 adducts per 10^8^ nucleotides, respectively [37]. In the following study, the authors investigated gastric mucosa samples collected from Chinese patients and Japanese ones and identified 7 LPO-related DNA adducts [39]. Among the 7 adducts, 1,*N*^6^-etheno-2′-deoxyadenosine (*ε*dA) and H*ε*dC were present in all 22 samples with extremely high levels (30–130 adducts per 10^8^ nucleotides). Although absent in some samples, H*ε*dA exhibited the highest level in a sample at ~3400 adducts per 10^8^ nucleotides. Interestingly, the adduct levels were able to be used to discriminate between the origins of the samples (China or Japan) [39]. In addition to the DNA adducts derived from LPO products, certain epigenetic modifications may also have potential as predicitve biomarkers. Ohnishi et al. discovered a reduction in the 5-hmdC levels in the stomach of gastric cancer patients compared to subjects without gastric cancer [98]. The reduction in the 5-hmdC levels was similarly observed in patients with urinary tract urothelial carcinoma (UTUC) and without UTUC [99].

### 4.2. The Identification of DNA Adducts as Biomarkers to Assess Exposure to Environmental Pollutants

Humans and other organisms are exposed to numerous exogenous compounds, many of which are carcinogens. DNA adducts in organisms in the environment, including animals, plants, and microorganisms, can be used as indicators to monitor environmental pollution. Kanaly et al. reported the screening for DNA adducts in a sphingomonad soil bacterium exposed to acrolein, a commonly used biocide in hydraulic fracturing processes [40]. Three putative DNA adducts, 3-(2′-deoxyribosyl)-5,6,7,8-tetrahydro-8-hydroxy-pyrimido [1,2-*a*]purine-(3*H*)-one, 3-(2′-deoxyribosyl)-5,6,7,8-tetrahydro-6-hydroxypyrimido [1,2-*a*]purine-(3*H*)-one, and a possible butanone-ethenoadenine adduct were discovered, among which the former two adducts were confirmed through synthetic standards [40]. The result indicates that it is likely that DNA adducts in soil bacteria are used to monitor the existence of environmental pollutants, although the study itself was not conducted with soil bacteria collected in polluted soils.

A recently published article attempted to link DNA adducts to the pollution load in situ through screening for adducts in wild populations of a Baltic sentinel species, the amphipod *Monoporeia affinis*, which were collected at 19 stations with different pollution loads in the Bothnian Sea and the Northern Baltic Proper of Sweden [100]. Most putative DNA adducts discovered exhibited positive correlations with the sediment concentrations of polycyclic aromatic hydrocarbons (PAHs), but both positive and negative correlations between the levels of adducts and metals (As, Co, Zn, Pb, Cr, and Hg) were observed [100]. Although the results of the study have not shown unambiguous associations between the levels of DNA adducts and the pollution loads due to the complex nature of DNA adducts and pollution, the study still demonstrates the likelihood that DNA adducts in environmental organisms are used as indicators for environmental pollution.

### 4.3. The Identification of DNA Adducts as Aging Biomarkers

The formation of DNA adducts is usually associated with carcinogenesis; however, DNA adducts are a form of DNA damage and can be related to other biological effects.

Aging is a complex and multifaceted process. Although the aging process is associated with an array of features at the molecular, cellular, and physiological levels, it has been proposed that DNA damage affects most, if not all, aspects of the aging phenotype and, as a result, can be considered to be a unifying cause of aging [101]. In this regard, the formation of DNA adducts may play a critical role in the aging process [102], because other types of DNA damage, such as strand breaks and depuration, largely stem from the formation of DNA adducts. Thus, DNA adducts have the potential to be used as aging biomarkers.

A recent article provides a good note for the point, in which Guilbaud et al. observed significant age dependence for multiple DNA adducts [103]. In the study, the authors employed an untargeted DNA adductomic approach that was similar to the original method developed by Kanaly et al. [8] to screen for DNA adducts in DNA extracted from rat and human tissues. It was found that DNA adducts were species-, tissue-, age-, and sex-dependent, and in particular, significant age-dependence for 36 adducts, including *N*^2^-carboxymethyl-dG (*N*^2^-CMdG), 5-hmdC, and 8-oxo-dG in rat, and 5-hmdC and *ε*dA in the human heart was observed. Significant age-dependent increases in the levels of 5-hmdC and *ε*dA were discovered in human heart tissue. The accumulation of the adducts correlates with loss of DNA repair activity, suggesting that the adducts may have the potential as aging biomarkers [103]. It is noted that 5-hmdC is the oxidation product of 5-mdC, an important epigenetic mark, and the age-dependence of 5-hmdC is echoed by a newly published report, which identified specific cytosines with methylation levels that change with age across numerous species [104].

### 4.4. The Identification of DNA Adducts to Assess Nanomaterial-Caused Damage

Although nanoparticles have natural sources, their toxicity has not caused concerns until the last 30 years. The toxicity of nanoparticles is dependent on complex interactions between the particles and specific biological microenvironments, and thus it is difficult to draw general conclusions. Nonetheless, a major nanotoxicity mechanism is the induction of oxidative stress and production of ROS [105], which can be assessed through ROS-induced DNA adducts.

The Totsuka laboratory made a global search for DNA adducts using the DNA adductomic approach in the lungs of mice exposed to nanosized magnetite [43]. Compared to the control, the samples from mice exposed to magnetite exhibited 27 additional putative DNA adducts, among which etheno-2′-deoxycytidine (*ε*dC), an inflammation- and oxidative stress-related adduct, was observed to highly correlate with magnetite exposure. The observation suggests that inflammatory response or oxidative stress might be involved in magnetite-induced genotoxicity in the murine lung [43].

In 2020, Qiu et al. similarly investigated DNA adducts in two environmentally relevant bacteria exposed to nanoscale lithium nickel manganese cobalt oxide (NMC), a cathode material in lithium-ion batteries [106]. Among the NMC exposure-induced DNA adducts, most resulted from oxidative stress and LPO. In the study, the induction of oxidative stress and generation of intracellular ROS were confirmed through using fluorescence probes.

### 4.5. The Identification of DNA Adducts as Exposure Biomarkers in the Determination of Exogenous Carcinogens Causing Cancers

The formation of DNA adducts may contribute to the etiology of cancers, and the structures of DNA adducts carry critical information on the structures of the reactive chemical species that directly attack DNA, consequently, the identification of DNA adducts plays a pivotal role in the exploration of the origin of cancers, particularly in the search for exogenous compoundscausing cancers [107].

The identification of the culprit to induce Balkan endemic nephropathy (BEN)-associated upper urothelial cancer (UCC) is an example of the central role of DNA adducts in the etiology of cancers. BEN is a familial chronic tubulointerstitial nephropathy that usually develops into UCC and is prevalent in some rural regions of the Balkan peninsula. It was described almost 70 years ago; however, the cause of BEN has remained unaddressed for approximately 50 years. Multiple exogenous factors for the BEN etiology have been hypothesized, including metals and metalloids, ochratoxin A, organic chemicals from Pliocene lignite deposits, and aristolochic acids (AAs), a group of naturally occurring compounds present in many plant species of the *Aristolochiaceae* family [108]. Finally, AAs are determined to be the primary causative agents for the etiology of BEN based on several different types of evidence, in which the discovery of AA-derived DNA adducts in renal tissues of BEN patients since 2002 provides a critical piece of evidence for exposure to AAs [108]. AA-DNA adducts were detected first through the ^32^P-postlabeling assay [109] and later by LC-MS [110].

A similar case is to search and identify the likely causative agent that contributes to the high incidence of esophageal cancer in certain regions of China [13]. Like the case of BEN, it has been known for many years that esophageal cancer is particularly prevalent in the regions surrounding the Taihang Mountains in northern China, but its etiology has remained largely unknown. It has long been suspected that *N*-nitrosamines, a class of compounds notorious both for the potent carcinogenicity of many of its members and for their widespread occurrence throughout the human environment [111], are an important etiological factor for esophageal cancer, however, definite evidence of a causal role has been lacking. Moreover, many different *N*-nitrosamines are present in the environment, further increasing the difficulty in the search for the potential culprits.

To address the issue, Totsuka et al. employed the DNA adductomic approach to make a global search for DNA adducts in tumorous and nontumorous tissues collected from subjects residing in areas with high and low incidences of esophageal cancer [13]. This led to the detection of many putative DNA adducts; through comparing the differences between the results obtained with the high- and low-incidence areas, an adduct that was highly specific to the high-incidence area was discovered. The adduct was structurally characterized as *N*^2^-(3,4,5,6-tetrahydro-2*H*-pyran-2-yl)-2′-deoxyguanosine (THP-dG) by using synthetic standards. THP-dG has been known to be formed from *N*-nitrosopiperidine (NPIP) via the cytochrome P450 metabolic activation pathway, and NPIP can induce tumors in the esophagus and liver of rats. In addition, the NPIP-induced mutational pattern in rats was similar to that in the samples from esophageal cancer patients. Considering that some vegetables and shallow well water contain higher nitrate contents in the high-incidence area than in the low-incidence one, and piperidine or its precursor piperine can be ingested via diet, it was thus speculated that NPIP might be formed endogenously by nitrosation of piperidine. Although it has not fully established a causative role of NPIP in the development of esophageal cancer in specific regions, the study provides critical clues for further investigations and demonstrates the power of DNA adductomics in the search for environmental culprits that may induce cancers [13].

Another case involves the determination of the structure of colibactin, a human gut bacterial genotoxin produced by organisms (e.g., *E. coli*) harboring the *pks* genomic island that probably contributes to the development and progression of colorectal cancer (CRC) [14]. In this study, the CNL-MS^3^ adductomic method run in an HR mass spectrometer was used to screen the DNA samples for DNA adducts. Two putative colibactin-DNA adducts were discovered, and their structures were speculated on the basis of the mass spectral data and were finally confirmed through synthetic standards. The identification of these DNA adducts and characterization of their structures provide critical evidence for the determination of the structure of colibactin and the molecular mechanism underlying its genotoxic effects, paving the way for elucidating the involvement of the genotoxin in the development or progression of CRC. Meanwhile, the DNA-colibactin adducts are potential biomarkers of colibactin exposure and cancer risk [14].

It should be noted that in the study, the DNA adductomic approach plays an irreplaceable role because all previous attempts to directly isolate and structurally characterize colibactin have been unsuccessful [14]. The difficulty is attributed to the fact that the genotoxic activity of colibactin is contact-dependent and not observed when cells are treated with *pks*^+^ *E. coli* culture supernatants or cell lysates. Moreover, colibactin may be labile and/or recalcitrant to isolation, because attempts to directly identify colibactin using comparative metabolite analyses have failed as well. Indeed, the structure of colibactin suggests that it is actually an intermediate that is formed from a precursor (precolibactin) [14].

An additional case, which is somewhat similar to the colibactin study, also involves the utilization of the DNA adductomic approach to search for DNA adducts of a gut bacterial genotoxin tilimycin, a tricyclic pyrrolobenzodiazepine generated by an opportunistic gram-negative bacterial species *Klebsiella oxytoca* that causes antibiotic-associated hemorrhagic colitis [112]. In this case, the identification of DNA-tilimycin adducts helps elucidate the MOA of tilimycin.

## 5. Perspective and the Future Directions

### 5.1. DNA Adductomics Has the Potential to Play a Central Role in Biological Studies

So far, DNA adductomics has not received much attention; the studies relating to the field have been quite limited. Currently, DNA adductomics is primarily utilized as a tool for the search of certain classes of DNA adducts, such as ROS- and LPO-related adducts, or those derived from exogenous compounds investigated. The discovery of these DNA adducts is used as evidence of exposure to specific compounds or the MOAs of exogenous compounds because biomarkers, particularly exposure biomarkers, are the most important role of DNA adducts to date. In the essential, the importance of DNA adductomics depends on the roles that DNA adducts play in biological processes.

It is likely that DNA adducts play more important roles in biological processes than simple exposure biomarkers and/or biomarkers related to cancer risks. As pointed out by Schumacher et al., DNA damage affects most, if not all, aspects of the aging phenotype and thus can be a potentially unifying cause of aging [101]. Actually, DNA damage may play a central role in almost all aspects of intracellular biological processes, such as differentiation, proliferation, signal transduction, etc., needless to say, transcription and apoptosis.

Although DNA damage includes multiple different types, such as strand breaks, adduct formation, mutations, chromosomal aberrations, etc., DNA adducts may be the primary and fundamental form of DNA damage because most DNA damage actually originates from adducts. For example, the repair or spontaneous hydrolysis of DNA adducts generates strand breaks; unrepaired DNA adducts or certain repair processes can lead to mutations. Thus, the formation of DNA adducts can be a universally underlying driving factor of a variety of pathophysiological processes.

In this regard, the global landscape of DNA adducts in a cell can be viewed as some kind of code that contains information on the status, future, and fate of the cell. Although DNA is the blueprint of life, pristine DNA virtually does not exist, because DNA is under constant attack by endogenous and exogenous reactive chemical species. Thus, it may be the dynamic combination and interactome of genome and adductome that are the real and actual blueprint of life. In some sense, the transcriptome, proteome, and metabolome can be considered the “products” of the genome and DNA adductome because they are the biological processes or molecules downstream of DNA and epigenetic modifications are classified as DNA adducts. Thus, these omics should be combined with genomics and DNA adductomics to obtain a better understanding of intracellular biological processes and mechanisms.

An additional note about the DNA adductome is its dynamic nature. DNA adducts are constantly formed and also are rapidly removed via the DNA repair machinery [113]. It is thus important that the roles that the DNA adductome plays in a variety of biological processes are examined with the dynamic nature being fully considered. Indeed, the attempts to examine the associations between the levels of DNA adducts and the status of cancers obtain only limited success in a study involving oral cell DNA adducts and the lung cancer risk [17] but are unsuccessful in another study involving gastric cancer [114].

### 5.2. The Likelihood to Use the Totality of DNA Adductome as a Metric

Currently, a major issue in DNA adductomics is that the large amounts of data generated cannot be effectively utilized. The issue arises because virtually in any sample, large numbers of putative adducts can be detected through untargeted screening; however, a vast majority of the putative adducts are unknown, and it is difficult, if not impossible, to speculate on their structures, even their elemental compositions can be determined by HRMS. For example, the Turesky laboratory detected more than 1000 DNA adducts in the tissues of patients with prostate cancer and bladder cancer, most of which have not been reported in the literature and are of unknown origin [115]. Very recently, the laboratory reported the detection of 3956 and 3381 unknown adducts in the kidney and liver of rats dosed with 13 carcinogens, respectively [116]. In the studies, only quite limited numbers of DNA adducts were identified and carefully investigated; some analyses (e.g., principal components analysis) were indeed performed on the data but produced little useful information.

Since it is not possible to determine the structures of a vast majority of DNA adducts, a different approach may be tested, that is, using the totality of the DNA adductome as a metric. The totality of DNA adductome can be expressed in the form of a map, i.e., the adductome map, which has been used by the Matsuda and the Totsuka groups since the first study to develop DNA adductomics [8,13,36,37,38,39,40,41,43,44]. Although they are usually presented as two-dimensional illustrations, the maps are actually three-dimensional. The adductome map of each genotoxin is probably unique (in particular, if the locations of DNA adducts are integrated into the map; see the discussion about DNA mapping in Section 5.3); if so, they can be used as a metric for assessing the genotoxic potential of toxic compounds. Actually, the adductome maps may also apply to non-genotoxins (e.g., nanomaterials), because the agents often induce the production of ROS and thus indirectly lead to changes in the landscapes of DNA adducts. In this way, it is likely that the adductome maps can be associated with other metrics, in addition to genotoxicity. Of course, to achieve this, new advanced algorithms must be developed. Artificial intelligence (AI) can be of great assistance in the issue [117].

### 5.3. Globally Determining the Locations of All DNA Adducts (DNA Adductomic Mapping) Should Be the Next Step in the Development of DNA Adductomics

At present, the LC-MS-based approach is virtually the sole technique for global detection of DNA adducts. Although it is highly sensitive and specific in the detection of DNA adducts, the technique loses information on the locations of adducts in DNA strands. The locations of DNA adducts in strands are considered to be a pivotal factor in their biological consequences [118]. Some studies have demonstrated that the distribution of DNA adducts (e.g., 8-oxo-dG) in strands is not even [119,120]. Thus, a truly complete landscape of DNA adducts must contain the locations of DNA adducts. Globally determining the locations of all DNA adducts can be termed “DNA adductomic mapping”. Similar notions such as “mapping DNA adductomics” have been proposed, although these notions mostly focus on specific types of DNA damage like 8-oxo-dG or strand breaks [120]. Despite the small differences in the meanings of the notions, it is clear that this is an important direction of DNA adductomics. The mapping of DNA damage has been reviewed extensively [120,121,122,123,124,125]. However, at present mapping of DNA damage is still limited to certain types of DNA damage or adducts, including single- and double-strand breaks, AP sites, uracil and ribonucleotides, 8-oxo-dG, UV-induced DNA damage [CPD and (6-4) pyrimidine–pyrimidone photoproducts], malondialdehyde- and benzo[*a*]pyrene-dG, and cisplatin-induced crosslinks, and the resolutions are usually at the levels of hundreds of bp [120]. The methods that can achieve single-nucleotide mapping of adducts within whole genomes have been scarce [119], and it is certainly a great challenge to achieve global mapping of all DNA adducts, particularly under high resolution.

### 5.4. SERS Is a Potentially Promising Technique for DNA Adductomic Mapping

For global detection of DNA adducts, SERS is a very attractive technique due to several advantages over LC-MS/MS-based techniques. First, SERS directly detects DNA adducts in intact DNA, thus removing the step of sample preparation and greatly lowering the artifacts, such as oxidation caused in the process of preparing samples. Second, SERS is much more sensitive and thus needs much smaller DNA amounts compared to LC-MS-based techniques (sub-nanogram vs. microgram). Third, SERS can determine the locations of DNA adducts; namely, it has the potential for mapping DNA adducts. Therefore, SERS has the potential for global mapping of DNA adducts. However, specialized algorithms or machine learning-based modeling may be necessary due to the fingerprint characteristics of the spectra obtained.

## 6. Conclusions

DNA adductomics has entered its second decade. Great advances have been made, and DNA adductomic screening has been routine as long as LC-MS/MS instrumentation is accessible. DNA adductomics has shown its value and power in the search for the genotoxins that contribute to the etiology of cancers by providing critical structural information about the genotoxins. Nonetheless, the field is still in its infancy, and there exist many opportunities for great advances. Combined with other omics techniques, DNA adductomics can offer researchers a better picture of the important issues concerning environmental exposures and human health.
